# L2 Selves, Control-Value Appraisals, and Engagement in AI-Supported Informal Digital Learning of English: An SEM and fsQCA Approach

**DOI:** 10.3390/jintelligence14070127

**Published:** 2026-07-01

**Authors:** Xueheng Zhou, Honggang Liu

**Affiliations:** 1 School of Foreign Languages, Yancheng Institute of Technology, Yancheng 224051, China; xhzhou23@ycit.edu.cn; 2 School of International Studies, Soochow University, Suzhou 215006, China

**Keywords:** AI-supported IDLE, learner engagement, L2 selves, control-value appraisals, SEM, fsQCA

## Abstract

This study examined learner engagement in AI-supported informal digital learning of English (IDLE) by integrating L2 selves and control-value appraisals within a Control-Value Theory framework. Drawing on data from 653 Chinese university students, the study employed structural equation modeling (SEM) to test the associations among ideal L2 self, ought-to L2 self, control appraisal, intrinsic value appraisal, extrinsic value appraisal, and learner engagement. It further used fuzzy-set qualitative comparative analysis (fsQCA) to identify configurational pathways associated with high engagement. The SEM results showed that both ideal L2 self and ought-to L2 self were positively associated with the three types of control-value appraisals, which were in turn positively associated with engagement. Mediation analysis indicated that control appraisal, intrinsic value appraisal, and extrinsic value appraisal mediated the associations between L2 selves and engagement. The fsQCA results revealed six configurations associated with high engagement, suggesting that engagement may be linked to multiple motivational-appraisal patterns. These findings highlight the importance of both average associations and configurational diversity in understanding AI-supported IDLE engagement.

## 1. Introduction

The rapid development of artificial intelligence (AI) has reshaped the ways university students engage in informal digital learning of English (IDLE) (J. S. Lee, 2026; H. Liu & Fan, 2026; G. Liu & Ma, 2024). With the support of chatbots, machine translation systems, intelligent writing assistants, speech recognition applications, and adaptive learning platforms, learners can now access immediate feedback, personalized practice, and interactive English learning opportunities beyond the classroom (H. Liu & Fan, 2025; Pack & Maloney, 2023; X. Wang et al., 2024). In the present study, the AI tool primarily used by participants was the Doubao large language model, which provides chatbot-based interaction and language support functions. These affordances have made AI-supported IDLE an increasingly important extension of formal English education (T. Lee & Cho, 2025; G. L. Liu et al., 2024). However, the availability of AI tools does not automatically lead to meaningful learning. In such open and self-directed environments, students may use AI tools superficially to complete tasks, or they may engage more deeply by experimenting with language, reflecting on feedback, and sustaining independent practice (H. Liu et al., 2025; Mohebbi, 2025). Therefore, learner engagement becomes a key issue in understanding how AI-supported IDLE contributes to English learning (Aljabr & Al-Ahdal, 2024; G. L. Liu et al., 2025). Examining engagement in this context can help clarify how students move from simple access to AI tools toward purposeful and sustained participation in English learning.

Despite growing attention to digital and AI-assisted English learning, learner engagement in AI-supported IDLE remains insufficiently explained. Much existing research has focused on technology acceptance, frequency of tool use, learner attitudes, or learning outcomes (G. L. Liu et al., 2025; G. Liu & Ma, 2024), but using AI tools does not necessarily indicate sustained or meaningful engagement. Less is known about why some students become more actively engaged in AI-supported IDLE than others. L2 selves may offer one explanation, as learners’ imagined future English identities and perceived obligations can be closely related to their motivation for English learning (J. S. Lee & Lee, 2021; X. Wang et al., 2026). However, L2 selves alone may not fully explain engagement, because students also evaluate whether AI-supported IDLE is controllable, enjoyable, meaningful, or useful. From a Control-Value Theory perspective, such appraisals may be important for understanding engagement. Yet few studies have integrated L2 selves, control-value appraisals, and engagement within the context of AI-supported IDLE. This gap limits a fuller understanding of the motivational and evaluative conditions associated with AI-mediated English learning engagement.

To address this gap, the present study draws on Control-Value Theory to examine the associations among L2 selves, control-value appraisals, and learner engagement in AI-supported IDLE. Based on data from 653 Chinese university students, this study first uses structural equation modeling to examine whether ideal L2 self and ought-to L2 self are positively associated with control appraisal, intrinsic value appraisal, and extrinsic value appraisal, and whether these appraisals are positively associated with engagement. It further examines whether control-value appraisals mediate the associations between L2 selves and engagement. In addition, fuzzy-set qualitative comparative analysis is used to identify different configurations of L2 selves and control-value appraisals associated with high engagement. By combining SEM and fsQCA, this study provides both a variable-oriented and a configurational account of engagement in AI-supported IDLE. Specifically, this study makes three main contributions. First, it integrates L2 selves with Control-Value Theory to extend motivational and appraisal perspectives in AI-supported digital language learning. Second, it examines control-value appraisals as key mediating mechanisms linking L2 selves to learner engagement, thereby clarifying the psychological processes underlying AI-supported IDLE. Third, it applies fsQCA to identify multiple configurational pathways to high engagement, highlighting the equifinal nature of learner engagement in AI-mediated contexts. In doing so, it offers a more comprehensive explanation of how motivational self-guides and appraisal processes jointly shape AI-mediated English learning engagement. Accordingly, this study is guided by the following research questions:

RQ1: How do L2 selves and control-value appraisals relate to learner engagement in AI-supported IDLE?

RQ2: What configurational combinations of L2 selves and control-value appraisals are associated with high learner engagement in AI-supported IDLE?

## 2. Literature Review

### 2.1. Engagement in AI-Supported Informal Digital Learning of English

IDLE refers to English learning activities that occur outside formal classroom instruction through digital resources and online environments (J. S. Lee & Lee, 2021). Unlike teacher-directed classroom learning, IDLE is usually characterized by learner autonomy, flexible access, authentic exposure, and personally selected learning activities (Y. Gao et al., 2025). With the rapid development of artificial intelligence, IDLE has become increasingly supported by AI-based tools such as chatbots, machine translation systems, intelligent writing assistants, speech recognition applications, and adaptive learning platforms (G. L. Liu & Zhao, 2025). These tools can provide learners with immediate feedback, personalized practice, multimodal input, and opportunities for independent interaction with English (G. L. Liu & Hossain, 2026). In AI-supported IDLE, students are no longer merely exposed to digital English resources; they can also receive interactive support and generate individualized learning experiences (Zou et al., 2025a). However, such openness also places higher demands on learners’ self-regulation, judgment, and sustained participation (G. L. Liu et al., 2025). Therefore, AI-supported IDLE should be understood not only as a technological learning context, but also as a motivationally and cognitively demanding environment.

Learner engagement is particularly important in AI-supported IDLE because the availability of AI tools does not necessarily guarantee meaningful learning (G. Yang & Du, 2025). Engagement reflects the extent to which students participate behaviorally, invest cognitively, respond affectively, and interact socially during learning activities (X. Wang & Reynolds, 2024). In AI-supported IDLE, behavioral engagement may involve regular use of AI tools for English practice, while cognitive engagement may include evaluating AI-generated feedback, revising language output, and applying learning strategies (Y. Wang & Guo, 2026). Affective engagement refers to learners’ interest, enjoyment, or emotional involvement, whereas social engagement may involve interaction with AI agents, peers, or wider digital communities (X. Wang & Wang, 2024). Since AI-supported IDLE often occurs outside direct teacher supervision, learners’ engagement becomes a key indicator of whether they are using AI tools actively and meaningfully (G. L. Liu et al., 2024). Thus, examining engagement in this context can help reveal how students move beyond simple technology access toward sustained and purposeful participation in AI-mediated English learning.

### 2.2. L2 Selves and Control-Value Appraisals in AI-Supported IDLE

L2 selves provide an important motivational lens for understanding why learners may engage differently in AI-supported IDLE (J. S. Lee & Lee, 2021). The ideal L2 self refers to learners’ desired future image as competent L2 users, whereas the ought-to L2 self reflects attributes that learners believe they should possess in order to meet external expectations or avoid negative outcomes (Jiang & Dewaele, 2015). In the context of English learning, the ideal L2 self may involve imagining oneself communicating confidently in English for academic, professional, or intercultural purposes (Jang & Lee, 2019). The ought-to L2 self, by contrast, may be connected with examination pressure, family expectations, institutional requirements, or future employment demands (J. S. Lee & Lee, 2021). In AI-supported IDLE, these self-guides may be associated with how students evaluate AI-mediated English learning (X. Wang et al., 2026). Learners with stronger future self-images or stronger perceived obligations may be more likely to view AI tools as relevant resources for improving English competence and approaching desired or expected future identities (Siridetkoon & Dewaele, 2018).

Control appraisal refers to learners’ perceived ability to manage, regulate, and complete learning activities (Shao et al., 2020). In AI-supported IDLE, control appraisal is especially important because students often learn outside direct teacher supervision and need to make independent decisions about their learning process (G. L. Liu et al., 2024). For example, they may need to select appropriate AI tools, formulate effective prompts, evaluate AI-generated feedback, revise language output, monitor learning progress, and avoid over-reliance on automated assistance (X. Wang et al., 2025). These demands make AI-supported IDLE a highly autonomous but also cognitively demanding learning environment (G. L. Liu & Zhao, 2025). When students perceive that they can manage these processes effectively, they may report stronger engagement in AI-mediated English learning. By contrast, when AI tools are experienced as difficult to control or when students feel uncertain about the quality of AI feedback, their engagement may be weakened (H. Liu, 2026). Thus, control appraisal represents a key evaluative condition for understanding learner engagement in AI-supported IDLE.

Value appraisals concern learners’ judgments about the importance, usefulness, and personal meaning of learning activities (C. Li et al., 2025). In this study, value appraisal is divided into intrinsic value appraisal and extrinsic value appraisal. Intrinsic value appraisal refers to the extent to which students perceive AI-supported IDLE as interesting, enjoyable, or personally meaningful (Chou, 2025). For instance, learners may find it engaging to communicate with AI chatbots, receive instant language feedback, or explore authentic English materials through digital tools (T. Zhao & Lian, 2024). Extrinsic value appraisal refers to the perceived usefulness of AI-supported IDLE for achieving external goals, such as improving academic performance, passing examinations, developing writing competence, enhancing employability, or preparing for international communication (J. Zhao & Yang, 2026). In the Chinese EFL context, extrinsic value should not be viewed simply as a less desirable form of motivation, because English learning is often closely linked to educational and career opportunities (H. Liu et al., 2025). Therefore, both intrinsic and extrinsic value appraisals may be meaningfully associated with learner engagement in AI-supported IDLE.

### 2.3. Control-Value Theory and the Present Study

Control-Value Theory provides a useful framework for understanding how learners evaluate and engage in achievement-related activities. According to this theory, learners’ emotional, motivational, and behavioral experiences are closely related to two major types of appraisal: control appraisal and value appraisal (Pekrun, 2006; Pekrun & Perry, 2014). Value appraisal can be further understood in terms of intrinsic and extrinsic value, where intrinsic value refers to the extent to which a learning activity is experienced as interesting, enjoyable, or meaningful in itself, whereas extrinsic value concerns its perceived usefulness for achieving academic, social, professional, or future-oriented goals (Pekrun et al., 2010). From this perspective, engagement is not only related to the availability of learning resources, but also to how learners evaluate their capacity to control learning and the value they attach to the learning experience (Shao et al., 2020). In this study, these appraisal processes are further extended to the context of AI-supported informal digital learning of English.

Control-Value Theory is particularly relevant to AI-supported IDLE because this learning context is highly autonomous, flexible, and technologically mediated (X. Wang et al., 2026). Unlike classroom-based English learning, AI-supported IDLE often requires students to make independent decisions about when, where, and how to learn (X. Wang et al., 2025). Students need to select suitable AI tools, formulate prompts, evaluate AI-generated feedback, monitor their progress, and decide how to integrate AI support into their English learning routines (Sun et al., 2025). These features make control appraisal especially important. At the same time, AI-supported IDLE may be evaluated differently by learners in terms of value. Some students may perceive AI-assisted English learning as enjoyable and personally meaningful because of its interactivity, immediacy, and personalized support (H. T. Li & Yan, 2026). Others may value it because it helps them achieve external goals such as better academic performance, stronger English competence, career preparation, or international communication (Z. Gao & Du, 2026). Therefore, Control-Value Theory offers a suitable lens for examining how students’ control and value appraisals are associated with engagement in AI-supported IDLE.

**Figure 1:**
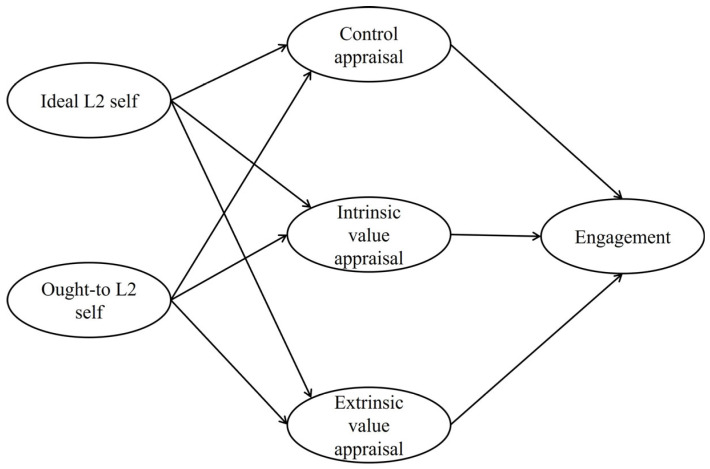
The proposed model.

Based on Control-Value Theory, the present study examines the associations among L2 selves, control-value appraisals, and learner engagement in AI-supported IDLE (see Figure 1), which illustrates the proposed conceptual model of the study. Ideal L2 self and ought-to L2 self are included as motivational antecedent variables that may influence how students evaluate AI-mediated English learning. Learners with a stronger ideal L2 self may perceive AI-supported IDLE as more controllable and valuable because it helps them move closer to their desired future English-using identity. Similarly, learners with a stronger ought-to L2 self may evaluate AI-supported IDLE as useful and manageable because it is associated with academic requirements, external expectations, or future responsibilities. In turn, control appraisal, intrinsic value appraisal, and extrinsic value appraisal are expected to be positively associated with learner engagement. In addition to testing these direct associations, this study further examines whether the three types of control-value appraisals mediate the associations between L2 selves and engagement. Based on Control-Value Theory, the following hypotheses are proposed:

**H1.**  *Ideal L2 self is positively associated with control appraisal in AI-supported IDLE.*

**H2.**  *Ideal L2 self is positively associated with intrinsic value appraisal in AI-supported IDLE.*

**H3.**  *Ideal L2 self is positively associated with extrinsic value appraisal in AI-supported IDLE.*

**H4.**  *Ought-to L2 self is positively associated with control appraisal in AI-supported IDLE.*

**H5.**  *Ought-to L2 self is positively associated with intrinsic value appraisal in AI-supported IDLE.*

**H6.**  *Ought-to L2 self is positively associated with extrinsic value appraisal in AI-supported IDLE.*

**H7.**  *Control appraisal is positively associated with learner engagement in AI-supported IDLE.*

**H8.**  *Intrinsic value appraisal is positively associated with learner engagement in AI-supported IDLE.*

**H9.**  *Extrinsic value appraisal is positively associated with learner engagement in AI-supported IDLE.*

**H10.**  *Control appraisal mediates the association between ideal L2 self and learner engagement in AI-supported IDLE.*

**H11.**  *Intrinsic value appraisal mediates the association between ideal L2 self and learner engagement in AI-supported IDLE.*

**H12.**  *Extrinsic value appraisal mediates the association between ideal L2 self and learner engagement in AI-supported IDLE.*

**H13.**  *Control appraisal mediates the association between ought-to L2 self and learner engagement in AI-supported IDLE.*

**H14.**  *Intrinsic value appraisal mediates the association between ought-to L2 self and learner engagement in AI-supported IDLE.*

**H15.**  *Extrinsic value appraisal mediates the association between ought-to L2 self and learner engagement in AI-supported IDLE.*

## 3. Method

### 3.1. Participants

A total of 653 undergraduate students were retained for the final analysis. Eligibility was restricted to students who were enrolled in undergraduate programs, had experience using AI tools in informal digital learning of English (IDLE), and voluntarily agreed to participate in the study. The data were collected from universities located in Jiangsu, Shaanxi, Guangdong, Hubei, and Jilin provinces in China. The detailed demographic characteristics of the participants, including gender, age, and disciplinary background, are presented in Table 1.

### 3.2. Instruments

A Chinese-language questionnaire was used for data collection. It included two parts: one addressing participants’ demographic characteristics and the other measuring the core variables in the context of AI-supported IDLE, including L2 selves, control-value appraisals, and engagement. Responses were provided on a five-point Likert scale, with anchors from 1 (strongly disagree) to 5 (strongly agree). All instruments were adapted to the AI-supported IDLE context following a structured modification procedure, including item wording revision, expert review, and a small-scale pilot test to ensure contextual appropriateness and clarity. The final version was further refined based on participant feedback before formal data collection.

#### 3.2.1. L2 Selves

L2 selves were measured using the scale developed by Papi and Abdollahzadeh (2012). The scale comprises two dimensions, i.e., ideal L2 self and ought-to L2 self, with eight items each. All items were carefully reworded to reflect AI-supported IDLE contexts (e.g., incorporating AI tools such as chatbots and writing assistants), and the adapted version was reviewed by three experts in applied linguistics before administration. A sample item is “I imagine myself using AI tools to communicate in English confidently with international friends or colleagues.” Cronbach’s alpha values were 0.896 and 0.902 for the two dimensions, respectively, and 0.899 for the overall scale. Confirmatory factor analysis (CFA) results showed a good model fit: *χ*^2^/*df* = 1.593, RMSEA = 0.030, NFI = 0.968, RFI = 0.962, IFI = 0.988, TLI = 0.986, and CFI = 0.988.

#### 3.2.2. Control-Value Appraisals

Control-value appraisals were measured using the scale developed by C. Li (2021). The scale includes three subdimensions: control appraisal, intrinsic value appraisal, and extrinsic value appraisal, comprising three, five, and five items, respectively. Minor wording adjustments were made to align the items with AI-supported IDLE settings, and the revised version was pilot-tested with a small group of students to ensure comprehension and contextual relevance. A sample item is “Achieving good grades in English, with AI-assisted IDLE, is very important to me.” Cronbach’s alpha values were 0.856, 0.866, and 0.859 for the three subdimensions, respectively, with an overall reliability of 0.866. CFA results showed a good model fit: *χ*^2^/*df* = 1.060, RMSEA = 0.010, NFI = 0.983, RFI = 0.979, IFI = 0.999, TLI = 0.999, and CFI = 0.999.

#### 3.2.3. Engagement

Engagement was measured using the scale adapted from Hoi and Le Hang (2021). The scale includes four dimensions, namely behavioral, cognitive, affective, and social engagement, with four items each. The original items were contextualized to reflect AI-mediated informal English learning activities, and minor revisions were made based on expert feedback to ensure alignment with AI-supported IDLE practices. A sample item is “I stay focused during AI-assisted IDLE activities.” Cronbach’s alpha values were 0.868, 0.876, 0.847, and 0.886 for the four dimensions, respectively, with an overall reliability of 0.918. In the structural equation model, learner engagement was specified as a second-order latent construct, with behavioral, cognitive, affective, and social engagement serving as first-order reflective dimensions. CFA results indicated a good model fit: *χ*^2^/*df* = 2.681, RMSEA = 0.051, NFI = 0.957, RFI = 0.948, IFI = 0.973, TLI = 0.966, and CFI = 0.973.

### 3.3. Data Collection

The survey was administered in April 2026 using a convenience sampling strategy. Instructors at several Chinese universities helped circulate the Wenjuanxing survey link to students. During data collection, standard ethical safeguards were implemented. Participants were first provided with information about the aims of the study, the voluntary nature of their participation, the confidentiality of their data, and their right to discontinue participation without any negative consequences. Only those who provided online informed consent were allowed to complete and submit the questionnaire. A total of 713 questionnaires were initially collected. After data screening, responses with identical answer patterns and completion times below 2 min were excluded, resulting in 653 valid responses for final analysis.

### 3.4. Data Analysis

Data were analyzed using SPSS 27.0, AMOS 26.0, and fsQCA 4.0. The analytic procedure proceeded in several stages. First, the dataset was examined for potential common method bias, followed by tests of distributional properties using skewness and kurtosis. Descriptive statistics and Pearson correlation analyses were then conducted to provide an initial overview of the variables and their bivariate associations. Second, the measurement model was evaluated to establish the reliability and validity of the instruments. Third, structural equation modeling (SEM) was used to assess the proposed direct relationships among the focal constructs. Fourth, mediation effects were tested through bootstrapping. Finally, fuzzy-set qualitative comparative analysis (fsQCA) was used to identify multiple configurational pathways associated with high engagement. Five condition variables were included in the fsQCA analysis: ideal L2 self, ought-to L2 self, control appraisal, intrinsic value appraisal, and extrinsic value appraisal, with engagement as the outcome variable. A direct calibration approach was employed to convert Likert-scale data into fuzzy-set membership scores. Specifically, following standard fsQCA practice for survey data, calibration anchors were defined based on the observed data distribution, where full membership was set at the maximum value (5), full non-membership was set at the minimum value (1), and the crossover point was set at the sample mean (Zhen et al., 2024). This approach allows calibration to better reflect the empirical distribution of the sample and reduces arbitrariness associated with fixed theoretical cut-off points. Consistency and PRI thresholds were set at 0.80 and 0.70, respectively, to determine sufficient configurations associated with high engagement. SEM and fsQCA were used for complementary analytic purposes. SEM was adopted to examine net effects and average associations among latent constructs, whereas fsQCA was used to explore whether high engagement could be associated with different combinations of motivational and appraisal conditions. Therefore, the fsQCA analysis was not intended to replicate the SEM results, but to provide a set-theoretic account of configurational equifinality.

## 4. Results

### 4.1. Results of Preliminary Analysis

To examine common method bias, Harman’s single-factor test was conducted (Harman, 1976). The results extracted nine factors with eigenvalues above 1, and the first factor accounted for 28.905% of the total variance, below the 50% criterion. Thus, common method bias was not a serious concern in this study. Table 2 presents the descriptive statistics and correlations. All mean values were above 3, with moderate standard deviations. Skewness and kurtosis values fell within the recommended ranges of ±2 and ±7, respectively, supporting univariate normality (Kline, 2023). All focal variables showed significant positive correlations, offering initial support for the proposed relationships.

### 4.2. Reliability and Validity Analysis

Table 3 reports the measurement model results. The four latent constructs showed acceptable reliability and validity, with composite reliability (CR) values above 0.70 and average variance extracted (AVE) values above 0.50, indicating adequate internal consistency and convergent validity (Hair et al., 2021). Discriminant validity was also supported, as each construct’s square root of AVE exceeded its correlations with the other constructs (Fornell & Larcker, 1981).

### 4.3. Results of SEM and Mediation Analysis

**Figure 2:**
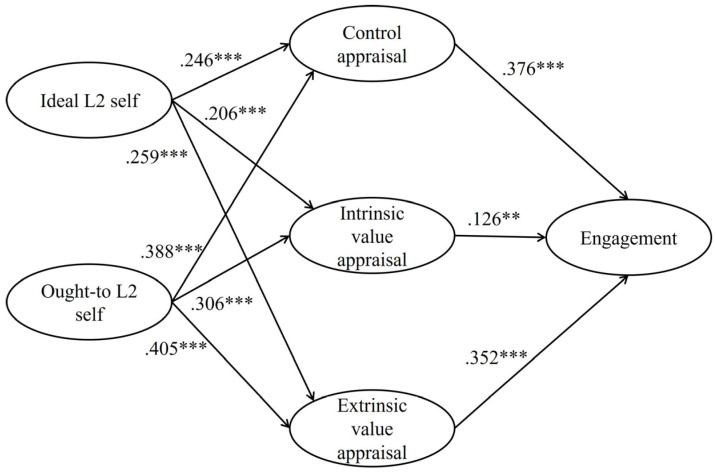
The final SEM model. Note: *** *p* < .001; ** *p* < .01.

The structural model showed an acceptable fit to the data: *χ*^2^/*df* = 1.397, RMSEA = 0.025, NFI = 0.921, RFI = 0.916, IFI = 0.976, TLI = 0.974, and CFI = 0.976. These results supported the adequacy of the proposed model. As shown in Table 4 and Figure 2, all proposed paths were significant.

Mediation was tested using 5000 bootstrap samples and 95% bias-corrected confidence intervals (CIs). As shown in Table 5, control appraisal, intrinsic value appraisal, and extrinsic value appraisal all showed significant indirect effects, with no CI including zero.

### 4.4. Results of fsQCA

Following common practice for calibrating Likert-scale data, the anchors for full membership, the crossover point, and full non-membership were set at 5, the mean, and 1, respectively (Zhen et al., 2024). Membership scores of 0.500 were adjusted to 0.501 to avoid exclusion from the fsQCA analysis (He et al., 2024). Before the configurational analysis, a necessity analysis was conducted for high engagement. A condition was considered necessary when its consistency exceeded 0.90 and its coverage was above 0.50 (Mo, 2026; Schneider & Wagemann, 2012). As shown in Table 6, the consistency values of all single conditions were below 0.90, indicating that no individual antecedent condition was necessary for high engagement. This suggests that high engagement is not driven by any single factor alone but by combinations of multiple conditions.

Following prior recommendations, the frequency threshold was set at 8 to ensure that the selected configurations covered at least 75% of the observed cases (Ragin, 2006; Patala et al., 2021). The consistency and PRI consistency thresholds were both set at 0.80 (Tan et al., 2025). The truth table was analyzed using fsQCA 4.0, and the intermediate solution was used as the main basis for interpretation, with the parsimonious solution consulted to identify core and peripheral conditions. The sufficiency results are reported in Table 7.

As shown in Table 7, six antecedent configurations led to high engagement. S2a and S2b were treated as sub-configurations because they shared the same core condition but differed in peripheral conditions. The overall solution consistency was 0.827, above the 0.80 threshold, indicating that these configurations constituted sufficient pathways to high engagement. The overall solution coverage was 0.888, indicating that the identified configurations covered a substantial proportion of the cases with high engagement (Fiss, 2011). In particular, ideal L2 self emerged as a core condition in several pathways, either independently or in combination with ought-to L2 self or intrinsic value appraisal. Meanwhile, intrinsic value appraisal, extrinsic value appraisal, and control appraisal also formed distinct pathways to high engagement under different conditions. Overall, these results highlight the configurational nature of engagement, suggesting that high engagement can be achieved through multiple combinations of L2 selves and control-value appraisals.

Robustness checks were conducted by adjusting key fsQCA parameters, following prior studies (Schneider & Wagemann, 2012). Specifically, the case frequency threshold was increased from 8 to 9 (Ye et al., 2024), and the consistency threshold was raised from 0.80 to 0.85 (Tan et al., 2025). As shown in Table 8, both adjusted analyses produced the same six configurations as the original solution, with identical consistency and coverage values. These results indicate that the identified configurations for high engagement were stable and robust.

## 5. Discussion

### 5.1. L2 Selves as Motivational Antecedents of Control-Value Appraisals

The SEM results showed that ideal L2 self was positively associated with control appraisal, intrinsic value appraisal, and extrinsic value appraisal in AI-supported IDLE. This finding suggests that students who reported a clearer and more vivid image of themselves as competent future English users also tended to report stronger perceptions of control, interest, and usefulness in AI-supported IDLE. In this context, AI tools may provide accessible resources through which learners connect present English learning activities with imagined future L2 identities. When students imagine themselves using English confidently for academic, professional, or intercultural communication, they may be more likely to perceive AI-mediated learning as manageable, meaningful, and relevant to their future development. This result is consistent with previous studies showing that ideal L2 self is closely related to learners’ motivation, learning effort, and engagement in language learning (J. S. Lee & Lee, 2021; X. Wang et al., 2026). More importantly, the present finding extends this line of research by suggesting that ideal L2 self may also be associated with learners’ control-value appraisals in AI-supported IDLE environments. The SEM findings should be interpreted as evidence of average associations among constructs, whereas the fsQCA findings should be understood as set-theoretic patterns of sufficient configurations within the present sample.

The results also showed that ought-to L2 self was positively associated with the three types of control-value appraisals. This finding is noteworthy because the role of ought-to L2 self has often been reported as weaker, more context-dependent, or less stable than that of ideal L2 self in previous L2 motivation research (C. Li et al., 2025; L. Yang & Li, 2026). In the present study, however, students with higher levels of ought-to L2 self also tended to report higher levels of control appraisal, intrinsic value appraisal, and extrinsic value appraisal. One possible explanation is that Chinese university students’ English learning is closely connected with external expectations, academic requirements, employment pressure, and social mobility. These externally imposed expectations may be internalized by learners as personally meaningful academic and career goals in the Chinese EFL context. In AI-supported IDLE, these internalized expectations may be interpreted by learners as pragmatic learning goals, which are associated with their tendency to view AI tools as useful resources for improving English performance and meeting future demands. Therefore, ought-to L2 self should not be understood simply as external pressure. Rather, in this context, it may be associated with socially shaped motivational orientations that are linked to students’ perceived control and value in AI-assisted independent English learning (X. Wang et al., 2026; J. Wang & Zhang, 2025).

### 5.2. Control-Value Appraisals as Mechanisms Linking L2 Selves to Engagement

The mediation results further revealed that control appraisal significantly mediated the associations between both L2 selves and engagement. Specifically, students with stronger ideal and ought-to L2 selves tended to report higher perceived control in AI-supported IDLE, which was in turn associated with higher levels of engagement. In highly open and flexible digital learning environments, students need to decide what tools to use, how to evaluate AI-generated feedback, how to manage learning time, and how to integrate AI support into their own English learning routines. When learners perceive these processes as controllable, they tend to report higher behavioral participation, cognitive investment, emotional involvement, and social interaction. This interpretation is consistent with control-value theory, which emphasizes the close relationship between perceived control, achievement emotions, and learning-related behavior (Pekrun, 2006; Pekrun & Perry, 2014). It also echoes research on self-directed and technology-enhanced language learning, where learner control and self-regulation are closely linked to engagement in digital learning contexts (Ghorbani & Golparvar, 2020; X. Wang & Wang, 2024).

Intrinsic value appraisal also served as a significant mediator between L2 selves and engagement, although its effect size was relatively smaller than that of control appraisal and extrinsic value appraisal. This result suggests that students who perceived AI-supported IDLE as interesting, enjoyable, or personally meaningful also tended to report higher engagement in such learning activities. When AI-mediated English learning is experienced as intrinsically valuable, learners may be more willing to explore authentic materials, interact with digital tools, and maintain attention during independent learning. This finding is in line with studies emphasizing the importance of interest, enjoyment, and positive learning experiences in L2 engagement (Cai & Liu, 2025; H. Liu & Wang, 2025). However, the relatively weaker indirect association through intrinsic value appraisal suggests that engagement in AI-supported IDLE may not be linked to enjoyment alone. For many university students, AI-assisted English learning is not merely a leisure-oriented activity, but is also closely related to academic achievement, practical language use, and future development. Therefore, intrinsic value needs to be understood alongside learners’ perceived control and instrumental value (Sun et al., 2026; X. Wang & Reynolds, 2024).

Extrinsic value appraisal was another important mediator linking L2 selves to engagement. The results showed that students who perceived AI-supported IDLE as useful for achieving external goals also tended to report higher levels of engagement. This finding is particularly meaningful in the Chinese EFL context, where English learning is often associated with examinations, academic advancement, employability, international communication, and access to wider educational opportunities. AI tools may be associated with learners’ perception that these external goals are more attainable by providing instant feedback, personalized practice, translation support, writing assistance, and opportunities for autonomous exposure to English. Thus, students who perceived AI-supported IDLE as relevant to academic and future-oriented outcomes were more likely to report stronger engagement. This finding is consistent with previous research showing that instrumental goals and pragmatic values are closely associated with L2 learning motivation in EFL contexts (G. L. Liu et al., 2024; Zou et al., 2025b). At the same time, it challenges a simplistic view that extrinsic value is necessarily less desirable than intrinsic motivation. In AI-supported IDLE, extrinsic value may be interpreted as an adaptive motivational orientation when it is connected with learners’ future selves and perceived learning opportunities (G. L. Liu et al., 2025; X. Wang & Wang, 2024).

### 5.3. Multiple Configurational Pathways to High Engagement in AI-Supported IDLE

The fsQCA results complemented the SEM findings by revealing multiple configurational pathways associated with high engagement in AI-supported IDLE. For ease of interpretation, the six sufficient configurations were labeled as S1, S2a, S2b, S3, S4, and S5. These labels refer to different combinations of motivational and appraisal conditions associated with high engagement, rather than to individual variables or hypotheses. The necessity analysis showed that no single condition reached the threshold for being necessary for high engagement, suggesting that high engagement was not linked to any isolated factor alone. Instead, the sufficiency analysis identified six configurations associated with high engagement, indicating that different combinations of ideal L2 self, ought-to L2 self, control appraisal, intrinsic value appraisal, and extrinsic value appraisal could correspond to similarly high engagement. This finding supports a configurational understanding of learner engagement, in which engagement is viewed as a complex outcome associated with interdependent conditions rather than with single predictors operating independently (X. Wang & Reynolds, 2024; X. Yang & Liu, 2024). It also shows that SEM and fsQCA answer complementary questions: SEM identifies average associations, whereas fsQCA reveals alternative motivational-appraisal patterns among different learner groups.

Several configurations highlighted the important role of ideal L2 self in high engagement. In S1, S2a, and S2b, ideal L2 self appeared as a core condition, either independently or in combination with ought-to L2 self and intrinsic value appraisal. These pathways may be interpreted as future-self-oriented configurations, suggesting that students with a strong imagined future English identity tended to report high engagement under different appraisal conditions. For example, S1 indicated that high engagement was associated with the presence of ideal L2 self even when extrinsic value appraisal was absent as a core condition. This suggests that, for some learners, a vivid image of becoming a competent English user may be closely linked to engagement even when external utility is not central. S2a and S2b further showed that ideal L2 self could be combined with ought-to L2 self or intrinsic value appraisal, indicating that future-oriented self-guides may coexist with social expectations or personal interest in different ways. This finding is consistent with previous research emphasizing the motivational relevance of ideal L2 self for sustained effort, learning intention, and engagement (H. Liu et al., 2022; Shao et al., 2020). The configurational results further suggest that ideal L2 self is not associated with engagement through one uniform pattern, but through several possible combinations with other conditions.

Other configurations suggested that high engagement could also be associated with appraisal-driven or compensatory patterns. In S3, intrinsic value appraisal was a core condition, while control appraisal and extrinsic value appraisal were absent as core conditions. This pathway implies that some students may report high engagement mainly when AI-supported IDLE is experienced as interesting, meaningful, or personally enjoyable, even when perceived control or external utility is not central. In S4, extrinsic value appraisal appeared as a core condition, whereas control appraisal and intrinsic value appraisal were absent as core conditions. This configuration suggests that, for some learners, the perceived usefulness of AI-supported IDLE for academic, career, or future-oriented purposes may be closely linked to engagement, even when intrinsic interest is not prominent. In S5, control appraisal emerged as a core condition, together with the peripheral presence of ought-to L2 self and the absence of extrinsic value appraisal. This pattern indicates that learners’ perceived ability to manage AI-supported IDLE may be associated with high engagement even when external value is not emphasized. These findings resonate with control-value theory, which highlights the importance of control and value appraisals in learning-related motivation and engagement (Pekrun, 2006; Pekrun & Perry, 2014). More importantly, they suggest a compensatory logic: when one condition is absent, other conditions may still be associated with high engagement if they form a coherent configuration. These configurations suggest that learner engagement may arise through different combinations of identity-, value-, and control-related beliefs.

### 5.4. Theoretical and Pedagogical Implications

Theoretically, this study contributes to the understanding of AI-supported IDLE engagement by extending Control-Value Theory to AI-supported informal language learning. Rather than treating engagement as a direct outcome of technology access or AI use, the study positions learner engagement as closely related to students’ control and value appraisals of AI-supported English learning. Specifically, the findings suggest that learners’ perceptions of control, intrinsic value, and extrinsic value are meaningfully associated with their engagement, which supports the relevance of Control-Value Theory beyond traditional classroom-based achievement contexts. In addition, by incorporating ideal L2 self and ought-to L2 self as self-related motivational beliefs, this study further shows how future-oriented L2 self-guides may be connected with learners’ appraisal processes in AI-supported IDLE. The combination of SEM and fsQCA also enriches this theoretical account. While SEM identified general positive associations among the focal constructs, fsQCA revealed multiple configurations associated with high engagement, suggesting that engagement can be understood through both average associations and diverse motivational-appraisal patterns.

Pedagogically, the findings suggest that teachers should view AI-supported IDLE not merely as a technological supplement, but as a motivationally structured learning space. First, teachers can help students develop clearer future English self-images by guiding them to connect AI-assisted learning with academic communication, intercultural interaction, and future career development. Second, students’ perceived control should be strengthened through explicit guidance on how to select AI tools, evaluate AI-generated responses, set learning goals, and monitor learning progress. Third, teachers should enhance both intrinsic and extrinsic value appraisals by designing tasks that are enjoyable, meaningful, and practically relevant. For example, AI-supported IDLE activities can be connected with authentic communication, writing practice, vocabulary expansion, and career-oriented English use. In this way, students may be more likely to experience AI-assisted informal English learning as manageable, valuable, and personally relevant, thereby supporting more sustained engagement.

## 6. Conclusions

This study examined the associations among L2 selves, control-value appraisals, and learner engagement in AI-supported IDLE among Chinese university students. The SEM results showed that both ideal L2 self and ought-to L2 self were positively associated with control appraisal, intrinsic value appraisal, and extrinsic value appraisal, which were in turn positively associated with engagement. The mediation analysis further indicated that the three types of control-value appraisals served as significant mediators in the associations between L2 selves and engagement. In addition, the fsQCA results revealed multiple configurations associated with high engagement, suggesting that engagement in AI-supported IDLE may be linked to different combinations of future self-guides and appraisal conditions. Taken together, these findings suggest that learner engagement in AI-supported IDLE is not only related to learners’ motivational self-images, but also to how they evaluate the controllability, meaningfulness, and usefulness of AI-assisted English learning.

Several limitations should be acknowledged. First, this study used a cross-sectional design, which means that the identified associations should not be interpreted as causal relationships. Future research could adopt longitudinal or experimental designs to examine how L2 selves, control-value appraisals, and engagement change over time in AI-supported IDLE. Second, the data were collected through self-report questionnaires, which may be influenced by social desirability bias and common method variance. In addition, self-reported engagement may not fully reflect learners’ actual behavioral engagement in AI-supported environments. Although common method bias was assessed using statistical techniques, it cannot be fully ruled out. Future studies could therefore triangulate questionnaire data with AI-generated learning logs, prompt histories, platform interaction records, or teacher observations to obtain a more comprehensive understanding of learner engagement in AI-supported IDLE. Third, the participants were Chinese university students, so the findings may not be directly generalizable to other educational levels or cultural contexts. Future research could compare learners from different countries, disciplines, or language backgrounds to further explore how AI-supported IDLE engagement varies across contexts.

## Figures and Tables

**Table 1:** Demographic Characteristics of the Participants.

Variable	Category	N	Percentage (%)
Gender	Female	386	59.11
	Male	267	40.89
Age	18–19 years	236	36.14
	20–21 years	268	41.04
	22 years or above	149	22.82
Discipline	Humanities/Social Sciences	286	43.80
	Science/Engineering	299	45.79
	Other disciplines	68	10.41

**Table 2:** Descriptive statistics and Pearson correlations.

Variable	1	2	3	4	5	6
1 Ideal L2 self	1					
2 Ought-to L2 self	0.361 **	1				
3 Control appraisal	0.333 **	0.411 **	1			
4 Intrinsic value appraisal	0.275 **	0.327 **	0.274 **	1		
5 Extrinsic value appraisal	0.347 **	0.431 **	0.359 **	0.370 **	1	
6 Engagement	0.449 **	0.441 **	0.437 **	0.300 **	0.430 **	1
M	3.685	3.540	3.712	3.379	3.507	3.643
SD	0.793	0.801	0.662	0.812	0.781	0.603
Skewness	−0.985	−0.829	−0.362	0.115	0.347	−0.266
Kurtosis	0.483	−0.019	0.162	−0.126	−0.404	0.543

Note: ** *p* < .01.

**Table 3:** Results of CFA.

Variable	CR	AVE	The Square Root of AVE and Correlation Coefficient Matrix					
			1	2	3	4	5	6
1 Ideal L2 self	0.897	0.521	**0.722**					
2 Ought-to L2 self	0.902	0.534	0.401	**0.731**				
3 Control appraisal	0.856	0.665	0.379	0.467	**0.816**			
4 Intrinsic value appraisal	0.866	0.565	0.314	0.371	0.319	**0.752**		
5 Extrinsic value appraisal	0.859	0.550	0.398	0.487	0.416	0.432	**0.742**	
6 Engagement	0.845	0.576	0.534	0.514	0.523	0.360	0.519	**0.759**

Note: Bold diagonal values indicate the square roots of AVE.

**Table 4:** Results of SEM.

Hypotheses	*β*	S.E.	C.R.	*p*	Results
Ideal L2 self → Control appraisal	0.246	0.041	5.455	***	Supported
Ideal L2 self → Intrinsic value appraisal	0.206	0.054	4.406	***	Supported
Ideal L2 self → Extrinsic value appraisal	0.259	0.047	5.754	***	Supported
Ought-to L2 self → Control appraisal	0.388	0.041	8.287	***	Supported
Ought-to L2 self → Intrinsic value appraisal	0.306	0.052	6.396	***	Supported
Ought-to L2 self → Extrinsic value appraisal	0.405	0.047	8.521	***	Supported
Control appraisal → Engagement	0.376	0.037	7.797	***	Supported
Intrinsic value appraisal → Engagement	0.126	0.026	2.958	.003	Supported
Extrinsic value appraisal → Engagement	0.352	0.033	7.283	***	Supported

Note: *** *p* < .001.

**Table 5:** Results of mediation analysis.

Path	*β*	SE	95% CI		*p*	Results
			Lower	Upper		
Ideal L2 self → Control appraisal → Engagement	0.092	0.023	0.053	0.143	***	Supported
Ideal L2 self → Intrinsic value appraisal → Engagement	0.026	0.014	0.006	0.060	.003	Supported
Ideal L2 self → Extrinsic value appraisal → Engagement	0.091	0.021	0.055	0.139	***	Supported
Ought-to L2 self → Control appraisal → Engagement	0.146	0.025	0.102	0.199	***	Supported
Ought-to L2 self → Intrinsic value appraisal → Engagement	0.038	0.017	0.012	0.078	.003	Supported
Ought-to L2 self → Extrinsic value appraisal → Engagement	0.143	0.025	0.099	0.200	***	Supported

Note: *** *p* < .001.

**Table 6:** Necessity analysis of single conditions for high engagement.

Condition	Engagement	
	Consistency	Coverage
Ideal L2 self	0.847	0.807
~ Ideal L2 self	0.600	0.767
Ought-to L2 self	0.822	0.800
~ Ought-to L2 self	0.627	0.777
Control appraisal	0.838	0.822
~ Control appraisal	0.678	0.785
Intrinsic value appraisal	0.783	0.815
~ Intrinsic value appraisal	0.679	0.779
Extrinsic value appraisal	0.801	0.824
~ Extrinsic value appraisal	0.668	0.776

Note: “~” denotes the absence of a condition.

**Table 7:** Configurations for high engagement.

Antecedent Condition	S1	S2a	S2b	S3	S4	S5
Ideal L2 self	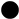	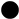	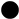			
Ought-to L2 self		●				●
Control appraisal				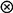	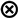	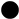
Intrinsic value appraisal			●	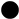	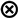	
Extrinsic value appraisal	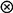			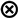	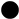	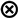
Consistency	0.889	0.891	0.900	0.943	0.949	0.933
Raw coverage	0.580	0.768	0.733	0.475	0.475	0.529
Unique coverage	0.020	0.035	0.010	0.007	0.013	0.011
Overall solution consistency	0.827					
Overall solution coverage	0.888					

Note: 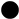 = presence of a core condition; 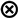 = absence of a core condition; ● = presence of a peripheral condition; 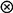 = absence of a peripheral condition; blank spaces indicate that the condition may be either present or absent.

**Table 8:** Robustness checks for configurations leading to high engagement.

Antecedent Condition	Adjusted Case Frequency Threshold						Adjusted Consistency Threshold					
	F1	F2a	F2b	F3	F4	F5	H1	H2a	H2b	H3	H4	H5
Ideal L2 self	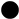	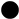	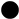				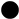	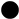	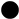			
Ought-to L2 self		●				●		●				●
Control appraisal				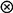	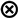	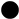				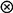	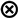	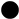
Intrinsic value appraisal			●	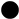	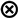				●	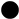	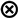	
Extrinsic value appraisal	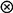			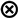	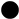	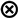	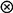			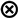	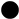	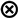
Consistency	0.889	0.891	0.900	0.943	0.949	0.933	0.889	0.891	0.900	0.943	0.949	0.933
Raw coverage	0.580	0.768	0.733	0.475	0.475	0.529	0.580	0.768	0.733	0.475	0.475	0.529
Unique coverage	0.020	0.035	0.010	0.007	0.013	0.011	0.009	0.035	0.010	0.007	0.013	0.011
Overall solution consistency	0.827						0.827					
Overall solution coverage	0.888						0.888					

Note: 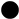 = presence of a core condition; 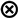 = absence of a core condition; ● = presence of a peripheral condition; 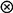 = absence of a peripheral condition; blank spaces indicate that the condition may be either present or absent.

## Data Availability

The raw data supporting the conclusions of this article will be made available by the authors on request.
